# Description of a Remarkable Species of Intestinal Vermes; Communicated to Dr.Miller

**Published:** 1803-12-01

**Authors:** James S. Stringham

**Affiliations:** Professor of Chemistry in Columbia College, and President of the New-York Physical Society


Description *of a remarkable Species of* Intestinal Vermes; *communicated to* Dr. Miller, *by* James S. Stringham, *M. D. Professor of Chemistry in Columbia College, and President of the New-York Physical Society.*

THE relation of occurrences, which from our reading and observation, we find seldom to have presented themselves to the eye even of the most experienced medical practitioners, is a duty incumbent as it is important. It is by this means that the limits of our inquiries are extended, and that we are prepared to encounter events, the existence of which we might never have suspected, till, by a fatal termination of the disease, dissection points out to us the presence of circumstances, of which, had we been previously aware, a different plan of cure would have been adopted, and the life of the patient probably preserved. This is a mode of acquiring experience truly humiliating; but how is it to be avoided, unless every physician, while he derives benefit from the observations of others, endeavours, at the same time, to contribute his mite towards the promotion of general knowledge, by a careful and faithful delineation of such particular occurrences as he conceives to be new and interesting? Under this impression I feel myself impelled to offer to your consideration the history of a case, the singular termination of which excited no small degree of surprise in all who witnessed it.

About nine months ago I was called upon to visit a lady in this city, who had been for some time in ill health. I found her somewhat emaciated. She had little or no appetite; complained of almost constant pain in the breast, near the xiphoid cartilage of the sternum; she had no cough; respiration was perfectly free and easy. I took from her a small quantity of blood, ordered a cooling purgative, and put her upon the use of the digitalis, with a light diet. This plan was persevered in for some time, till she took forty drops of the tincture, three times a day; beyond this it affected her head and stomach. Finding, however, that this did not produce the desired effect, I added a blister, which I kept discharging for several weeks. By this her complaints were so much relieved that I considered any further attendance as unnecessary. My patient went out of town, and I heard no more of her for several months, when she again sent for me. She informed me that she had continued free from her pain for a considerable time after the blister had healed, but that it had again returned, and, as she thought, with increased violence. The complexion was pallid; her pulse weak and irregular; her appearance much more emaciated than when I saw her last. I again put her on the same treatment, and again the blister relieved her pain: the suspension however proved but temporary; her former symptoms returned; in addition to which she also complained of great pain and soreness in the abdomen. I treated her upon the plan generally adopted where such indications exist, but without the least success; the pain in the abdomen particularly became extremely urgent, and she frequently told me, that when riding the smallest irregularity of motion in the carriage produced sensations almost insupportable. Her stomach had become so irritable, that every species of food was rejected as soon as swallowed; a constant tenesmus supervened, vened, although she voided no fæces unless assisted by cathartics or injections.

Finding every effort to relieve my patient ineffectual, that on the contrary her disorder appeared to be increasing, and her strength rapidly declining, I began to apprehend that death alone could alleviate her sufferings: I therefore entirely confined myself to palliatives, the principal of which was laudanum. She continued in this miserable state of existence several weeks, during which time the only moments of ease that she experienced were those passed in insensibility. On one of my morning visits to her, while in this situation, she told me that she was convinced in her own mind, that her complaints were kept up by some animal within her; that she could distinctly perceive its motion, particularly if she fasted for any length of time, and that she was compelled, by the uneasy sensations it produced, in her stomach, to endeavour to take food although she had no appetite for it, after which she felt greatly relieved. Supposing that her mind, enfeebled by the long-continued sufferings of her body, had become impressed with some of those incredible fictions, which report snakes and different kinds of reptiles to have become inhabitants of the human system, I at first ridiculed the idea; but finding that she was obstinately pertinacious of her opinion, I was under the necessity of promising to give some medicine which would dislodge the animal, should any such exist. For this I gave her a bitter infusion to be taken in the morning early, and at night a drastic purgative; which plan she was to continue every second day till she had taken three doses of each. I called to see her on the morning after she had taken the second dose: I was surprized to find her appearance uncommonly cheerful, and at her first salutation to me, "Doctor, I shall yet get well." On inquiring into the cause of this sudden change, her husband informed me that she had voided a considerable number of animals, of what kind he could not pretend to say, as he had never before seen any thing like them. The pot was shown me, in which I discovered a number of substances which I did not hesitate to pronounc animalcular. The nurse informed me that the medicine had operated very severely on my patient, and that she still continued to experience its effects. I requested her to convey another pot into the room, telling her that I should remain some time below stairs, and that if the medicine should again operate while I continued in the house, it was my wish to see what was evacuated. I had not waited long before my curiosity was gratified; scarcely any fæces were to be seen; the excrements consisting almost entirely of these substances, some of which I had put into a bottle filled with spirits, in order to examine them more particulatly at my leisure. They were about three fourths of an inch long, and to the inferior part of the body of each were attached a considerable number of legs. On first view I thought they were separate parts of several large worms, and that what appeared like legs, were nothing more than the different laminæ of filaments of the skin, separated by maceration. But, on a closer examination, I rejected the opinion, because the back of these animals did not appear to be covered by skin, but by a firm cartilaginous substance; there was likewise an uniformity of appearance and structure, which is not observed in filaments of the skin thus separated, viz. they were vesicular, each resembling a distinct sack, distended with a fluid, colourless and transparent. For my own part I have very little doubt that they belong to the order of vermes molusca, and to that genus termed actinia. I consider those vesicles as tentacula or legs, by which they adhere to different substances while feeding on their prey.

That this particular species of worms should be found residents of the human body, is, so far as my reading extends, entirely novel. Considering it a duty to do all in my power to aid the laudable efforts of yourself and colleague for the diffusion of medical information, I submit the above case to your inspection, with liberty to make such use of it as you may think proper.

Yours, &c. JAMES S. STRINGHAM.

P. S. Since writing the above I have met with a case, described in the sixth volume of the Transactions of the Royal Irish Academy, by Dr. Samuel Crumpe, which bears considerable analogy to that which I have just related to you; so much so, that I suspect the worms to be of the same genus, but can by no means agree with him in his speculations regarding their origin. These animals are found frequently floating in different kinds of waters, and, from experiments, many of them have been proved to resemble the polypus, in its powers of being multiplied by cuttings. May it not then easily be conceived, that some fragments of them should be swallowed with the water which these two patients drank, and that by adhering to the coats of the stomach and bowels, they should have become so numerous as to produce the effects already described. It may be proper to add, that since the voiding of these worms, the health of my patient has been perfectly restored, and that she has had no return of her complaints.

